# Chinese adolescents' experiences of performance confidence and anxiety and the role of instrumental teacher support

**DOI:** 10.3389/fpsyg.2026.1881466

**Published:** 2026-08-03

**Authors:** Yaxin Zhang, Naomi Norton

**Affiliations:** Centre for Music Education and Human Flourishing, School of Arts and Creative Technologies, University of York, York, United Kingdom

**Keywords:** adolescent musicians, Chinese instrumental learning, confidence-building, help-seeking behaviors, instrumental teacher support, music performance anxiety

## Abstract

**Introduction:**

This study explores adolescents' experiences of performance confidence and anxiety, particularly focusing on their perceptions of instrumental teacher support in relation to confidence-building and anxiety management in the Chinese context.

**Methods:**

A total of 144 adolescents aged 12–18 completed an online survey that included the Kenny Music Performance Anxiety Inventory for Adolescents (K-MPAI-A) and open-ended questions regarding help-seeking behaviors and perceived instrumental teacher support.

**Results:**

Most adolescents had experienced performance-related worry, nervousness, or anxiety, and over half of those who had such experiences reported engaging in help-seeking and perceived at least some level of instrumental teacher support for building confidence and managing anxiety. Results of the K-MPAI-A indicate low to moderate overall scores in this sample with cognitive concerns scoring higher than the other dimensions. Older age was positively associated with higher K-MPAI-A scores, whereas adolescents' perceptions of instrumental teachers providing support were negatively associated with reports of K-MPAI-A scores. Self-reported music grades were also negatively associated with K-MPAI-A scores indicating a significant group difference showing that beginner to intermediate pupils reported higher scores than those who classified themselves as advanced to highly advanced. Open-ended responses indicate that adolescents' help-seeking behaviors are shaped by perceived need, trust in teachers' expertise, teacher-student relationships, and concerns about judgment or embarrassment. The types of teacher support reported include cognitive management skills, preparation and practice, physical relaxation, and emotional encouragement.

**Discussion:**

The findings highlight the role of instrumental teachers in supporting adolescents' performance confidence and anxiety management in the Chinese instrumental learning context.

## 1 Introduction

Music Performance Anxiety (MPA) has been described as a persistent form of anxious apprehension related to musical performance that may be shaped by biological or psychological vulnerabilities as well as previous anxiety-related performance experiences, involving affective, cognitive, somatic and behavioral symptoms that may or may not affect performance quality (Kenny, 2009). MPA is reportedly widely experienced by musicians across the lifespan; however, there is still little research on MPA in adolescents (Kenny, 2011; Osborne and Kenny, 2005), particularly within the Chinese context (Zhu, 2020). Adolescence is a period of significant emotional, cognitive and social change, alongside a range of developmental challenges that may increase young people's vulnerability to stress and anxiety (Bolton, 2018; Compas et al., 2001; Salam et al., 2016). Early MPA research focused mainly on adult and professional musicians; however, a few studies have indicated that MPA can begin much earlier in musical development (Osborne and Kenny, 2005; Osborne et al., 2005; Tardif et al., 2024; Ryan et al., 2021). MPA levels among adolescent learners are likely to be shaped by a combination of individual characteristics, task-related factors, and the performance environment, highlighting the need to examine MPA in relation to both personal and learning-context variables (Papageorgi, 2022). Its development and persistence during adolescence are likely to be shaped by individual, interpersonal and contextual factors (Ananias de Lima et al., 2024).

Studies with young musicians indicate that MPA can begin early in a musician's development and may be associated with self-evaluative concerns, negative performance experiences, and concerns about mistakes (Kenny and Osborne, 2006; Osborne and Kenny, 2008; Patston and Osborne, 2016). Research to date (Osborne and Kenny, 2005; Dempsey and Comeau, 2019; Papageorgi, 2022) has indicated that older adolescents tend to report higher levels of MPA, which may relate to developmental changes during adolescence (including greater self-evaluation and awareness of others' judgement) as well as the task-related and environmental demands surrounding musical performance (including more challenging repertoire, formal assessments, auditions, and public performance). In some of the only research focusing on the Chinese context, Chan (2011) examined 174 Hong Kong music students aged 7 to 18 and found that pupils aged 11 to 18 reported slightly more MPA symptoms than those aged 7 to 10, though they did not find statistically significant age differences in their sample. Their findings further suggest that self-esteem and anticipated performance outcomes may help explain why MPA becomes more salient with age.

Differences relating to gender and/or sex have been widely examined but findings are inconsistent. Several studies with young or adolescent musicians have reported higher levels of MPA among girls/female students than boy/male students (Gül et al., 2026; Osborne et al., 2005; Osborne and Kenny, 2005; Papageorgi, 2022; Thomas and Nettelbeck, 2014). Papageorgi (2022) also found that girls experienced higher levels of MPA than boys; however, when gender was entered into a hierarchical regression alongside other variables, it was not a significant predictor of MPA. This may be explained by studies indicating that women/female musicians have a greater vulnerability to key correlates of MPA, including lower self-concept, reduced self-efficacy, and increased sensitivity to self-exposure and audience evaluation (Essau et al., 1999; Papageorgi, 2022). However, this pattern is not consistent across all studies: for example, Dempsey and Comeau (2019) and Ryan (2005) found no clear gender effect, and recent research in Chinese education contexts has also reported non-significant gender differences or associations (Cui et al., 2024; Ou and Qin, 2025). This suggests that gender may not operate as a straightforward predictor of MPA, though these Chinese studies focused on older learners rather than adolescents.

In terms of learning-related factors, instrumental examination grade level may contribute to adolescents' MPA levels, with higher examination grades linked to higher levels of MPA (Papageorgi, 2007), possibly reflecting greater performance demands and perfectionistic tendencies associated with higher levels of musical achievement (Butković et al., 2022; Patston and Osborne, 2016). Instrument type may also shape how young musicians experience MPA, though findings are not consistent across studies: for example, Yagisan (2009) found no major differences between instrument groups among Turkish adolescents whereas Kaspersen and Götestam (2002) found that Norwegian pianists and string players reported higher anxiety levels than students playing other instruments and Tardif et al. (2024) found that violinists experienced more anxiety than cellists. Moreover, performance format is likely to affect musicians' experiences with higher MPA levels reported in solo performance contexts compared with ensemble settings (Brugués, 2011; Casanova et al., 2018). Solo performance has been suggested to be the most stressful context for musicians, followed by small and ensemble performances (Papageorgi et al., 2013; Tardif et al., 2024). These findings suggest that demographic characteristics and learning-related variables are important considerations in research on adolescents' experiences with anxiety and confidence during musical performance.

Previous research suggests that music teachers play an important role in how young musicians experience confidence and MPA. Over a decade ago a large sample of instrumental and vocal teachers in the UK surveyed by Norton (2016) explicitly referred to their beliefs and behaviors relating to enhancing pupils' confidence (commonly linked to providing a supportive and psychologically safe learning environment) and helping pupils to avoid feeling anxious (often with reference to effective performance preparation strategies across five categories including teacher-centered approaches, stagecraft, physical exercises, mental approaches and attitudes, and the creation of performance opportunities). Similarly, Osborne (2015) suggests that building confidence requires practical and psychological preparation, such as secure musical preparation, realistic attitudes toward mistakes, positive self-talk, relaxation, imagery, performance simulation and low-pressure performance opportunities. Prior studies indicate that students value emotional support from their teachers (Studer et al., 2011), express a desire for more guidance on managing MPA (Tahirbegi, 2022), and may rely more on their teachers or parents for emotional support than adults who are likely to have a wider support network (Ananias de Lima et al., 2024). According to the social support framework (Malecki and Demaray, 2003), teacher support comprises several interrelated dimensions, including informational, instrumental, emotional and appraisal support. In the context of music learning and education, teachers are increasingly recognized as potentially important sources of social support for students experiencing MPA.

Previous studies have identified a variety of strategies that music teachers are able to apply to support their students in building confidence and/or managing MPA effectively, including strategies related to breathing, diet, focused attention, imagery, open discussion, physical activity, positive outlook, pre-performance routines, preparation, psychological performance skills, repertoire, safe environment, self-talk, simulated performance, suppression and visualization (see Gill et al., 2024; Huang and Yu, 2022; MacAfee and Comeau, 2023; Mazzarolo et al., 2023; Moura and Serra, 2021; Norton, 2016; Sieger, 2017; Tahirbegi, 2022). In 2021, Ryan and colleagues reported that almost half of the students surveyed shared that they received advice regarding MPA management from their teachers, but much of the advice did not effectively help students manage or was not explicit to cope with MPA. Many students are willing to receive support in MPA management from their teachers (Studer et al., 2011), but some students still feel uncomfortable disclosing their anxiety to them (Tahirbegi, 2022). These studies highlight the importance of relational conditions in help-seeking, meaning that teacher support needs to be not only available but also perceived by students as safe, approachable, and emotionally supportive.

As Herman and Clark (2023) identify, and the above literature review demonstrates, the presence of MPA has previously been investigated in a predominantly pathological framework with interventions designed primarily to help musicians to avoid unwanted symptoms. Within disciplines focusing on the health and wellbeing of musicians, there is a strong move away from a pathogenic paradigm to a salutogenic one that focuses on factors promoting wellbeing and health (Lubert et al., 2025). In response to this shift, the current study builds on existing research focusing on “managing MPA” but widens the discourse with adolescents and their teachers to include a more salutogenic model of “enhancing confidence” as well. Performance confidence is often discussed through related constructs such as self-efficacy, perceived competence or self-belief and generally refers to an individual's belief in their ability to perform successfully under evaluative conditions (Bandura, 1997). Musicians' confidence in their ability to perform may influence whether they interpret performance-related arousal as facilitative or debilitating rather than treating such arousal as inherently problematic (Hanton et al., 2004; Herman and Clark, 2023). Thus, building confidence in musical performance may help young musicians improve their ability to perceive an anxious response to stressors in the performance environment as facilitators of good outcome (Osborne, 2015). Research also found that MPA was negatively associated with self-efficacy among young musicians, suggesting the relevance of confidence-related factors in understanding adolescent MPA (Bersh, 2022; Dempsey and Comeau, 2019; Wang and Yang, 2024). More recently, Gill et al. (2026) found that a 14-week teacher-guided self-efficacy programme embedded in adolescent class and studio music lessons enhanced performance self-efficacy and was associated with improvements in psychological performance skills and music performance outcomes, with some reductions in anxiety, which provides further support for complementing MPA management with the proactive development of performance confidence.

Thus far, researchers have focused on theoretical approaches or teachers' perspectives on anxiety management for adolescent musicians (e.g., Gill et al., 2024; MacAfee and Comeau, 2023; Moura and Serra, 2021; Sieger, 2017) with comparatively less attention given to adolescents' experiences. Moreover, previous research has examined teachers' roles in students' performance preparation and MPA management (MacAfee and Comeau, 2023; Mazzarolo et al., 2023; Sieger, 2017), but limited research has focused specifically on adolescents' own perceptions of instrumental teacher support in this area, though Ryan et al. (2021) explored adolescent pianists' experiences of performance preparation, anxiety, and teacher support. Researchers in the following countries have documented performance anxiety among adolescent musicians in culturally specific educational contexts: Australia (Osborne and Kenny, 2005, Kenny, 2008; Thomas and Nettelbeck, 2014), Cyprus and the United Kingdom (Papageorgi, 2022), Germany (Fehm and Schmidt, 2006), Portugal (Dias et al., 2023), Romania (Sârbescu and Dorgo, 2014), Turkey (Dogan and Palanci, 2015; Gül et al., 2026), and the United States (Bersh, 2022). Research with adolescents in China remains rare (see Wang and Yang, 2024 for an exception) but the Chinese instrumental learning context offers an important setting for this inquiry because research with Chinese music students has linked autonomy-supportive teaching, positive teacher-student relationships and perceived social support with students' wellbeing, self-efficacy, confidence and achievement, and help-seeking intentions in relation to MPA (Jiang, 2026; Sun et al., 2026; Wang and Yang, 2024; Xu and Li, 2025). This suggests that confidence and anxiety in performance are not only individual psychological experiences but are also likely to be shaped by the relationships and learning environments in which students develop as musicians. However, existing evidence is still largely based on university-level music students: less is known about how adolescent instrumental learners make sense of anxious responses and confident feelings, and whether they perceive their instrumental teachers as approachable and helpful sources of guidance, reassurance and support.

Addressing this gap, the present study investigates adolescents' experiences of performance confidence and anxiety while studying in mainland China and their perceptions of how instrumental teacher support is experienced in relation to confidence development and anxiety management. Three research questions are formulated to achieve this aim:

- What are the scores and dimensional characteristics of K-MPAI-A among adolescents studying musical instruments in China, and how are these associated with demographic and learning-related factors?
- How does perceived instrumental teacher support relate to adolescents' self-reported K-MPAI-A scores and help-seeking behaviors?
- How do adolescents describe instrumental teacher support in relation to their confidence and/or anxiety management during musical performance?

## 2 Methods

### 2.1 Ethical considerations

Ethical approval for this study was granted by the School of Arts and Creative Technologies Ethics Committee at the University of York. The study also received Data Protection Impact Assessment (DPIA) approval from the University of York Data Protection Team before data collection began. Ethics materials (information sheets and consent forms) for both adolescents and parents/carers were created in English and translated into Chinese (Mandarin) by the first researcher. Informed consent was obtained from adolescents and parents/carers (for those under 18 years old) ensuring that they fully understood the purpose of the study, the procedures involved, and their right to anonymity, confidentiality, and withdrawal from the study. Pseudonyms, codes or respondent numbers have been used to avoid the likelihood of respondents being identifiable. For respondents under 18, written parental or carer consent was required prior to participation: parents/carers who provided consent received a one-time use survey link and were asked to share it with their child, while respondents aged 18 received the link directly. Respondents were informed in Mandarin at the beginning of the survey and on consent forms that the researcher is not medically qualified and that healthcare advice would not be provided; however, information about where to access support or guidance was included.

### 2.2 Recruitment and respondents

Recruitment for this survey study took place between April and September 2025, until the target sample size of about 150 respondents was achieved. This sample size was considered both feasible and sufficient to support meaningful statistical analysis. It was also informed by previous studies applying translated versions of the K-MPAI-A with similar sample sizes (see Dias et al., 2023; Sârbescu and Dorgo, 2014; Tardif et al., 2024). Respondents were recruited through Chinese art training institutions, schools, and organizations with which the first researcher had prior connections using a poster and online recruitment form with information about the study. All respondents confirmed their voluntary participation at the beginning of the study, were informed that they could stop completing the survey at any point and were provided with a user-specific randomly generated code allowing them to request withdrawal of their data while preserving anonymity. A total of 153 eligible respondents aged 12-18^1^ accessed the survey. Of those 144 completed the questionnaire and were included in the final analysis. All respondents were born and are living in mainland China. Table 1 demonstrates the demographic characteristics of respondents included in the final analysis.

**Table 1:** Gender distribution by school level among respondents included in the analysis (*N* = 144).

School level	Boys	Girls	Others/prefer not to disclose	Total
Primary school	6	8	0	14
Junior high school	26	33	7	66
Senior high school	21	31	8	60
Not reported	2	2	0	4
Total	55	74	15	144

### 2.3 Instruments

The survey was distributed online via Qualtrics survey software. Screening questions were included at the beginning to ensure eligibility. For example, respondents were asked whether they were currently learning a musical instrument with at least one teacher; only those who selected “Yes” were allowed to proceed, while others were excluded. Skip logic was applied throughout the survey to enhance its internal flow and ensure question relevance.

Self-reported performance-related anxiety and arousal responses were assessed by applying the Kenny Music Performance Anxiety Inventory for Adolescents (K-MPAI-A; Osborne and Kenny, 2005). This scale involves 15 items assessing adolescents' somatic, cognitive and behavioral responses to music performance. Each item is rated on a 7-point Likert scale, ranging from 0 (not at all) to 6 (all of the time), with higher scores indicating more frequent self-reported anxiety-related responses to musical performance. The present study refers to lower and higher K-MPAI-A scores rather than low and high levels of MPA. Responding at a low level on the index does not establish that an individual is experiencing MPA and may instead reflect normal performance-related arousal.

Perceived instrumental teacher support was assessed using study-specific survey items rather than a validated teacher-support scale. Respondents who reported experiencing nervousness, worry, or anxiety in performance were asked whether they had sought help from their instrumental teacher, with response options ranging from “Always” to “Never”. All respondents were also asked whether their instrumental teacher had provided guidance to support their confidence in performance, with response options of “Yes”, “Maybe”, and “No”. These items captured adolescents' self-reported perceptions of teacher support rather than direct evidence of teachers' actual practices.

The survey was designed in both English and Chinese (Mandarin). Permission to translate the K-MPAI-A^2^ using a translation-back translation method (Wild et al., 2009) to ensure linguistic accuracy and conceptual equivalence^3^ was obtained from Professor Dianna T. Kenny. A pilot study was conducted with three pupils (aged 12, 16, and 18) to assess the clarity, feasibility, and appropriateness of the survey (Williamon et al., 2021). Feedback indicated that instructions and items were clear, survey length was appropriate, and there were no technical issues. One ambiguity was noted in a question regarding the duration of formal lessons, which was subsequently revised and reordered to improve clarity. Minor procedural adjustments were also made, including adding a reminder and setting a link that could be used only once to prevent duplicate submissions.

### 2.4 Data analysis

This study uses a mixed-methods approach, integrating quantitative and qualitative analyses to explore adolescents' experiences of confidence and anxiety in musical performance alongside their perceptions of instrumental teacher support. Quantitative data have been analyzed using IBM SPSS Statistics (Version 31.0.1.0), including both descriptive and inferential statistics. All open-ended responses were provided in Mandarin and translated into English by the first author, a native Mandarin speaker, before analysis. Translation focused on preserving the semantic and contextual meaning of respondents' accounts. The English translations were analyzed using thematic analysis following Braun and Clarke (2006). Initial codes were developed through repeated reading of the translated responses and were then developed into broader themes and sub-themes. The English translations were checked against the original Mandarin responses throughout coding, theme development, and the selection of illustrative quotations to minimize potential loss or distortion of meaning.

Descriptive statistics are used to summarize respondents' demographic characteristics (e.g., age and gender) and K-MPAI-A scores. Frequencies and percentages have been calculated for categorical variables (e.g., self-reported music grade, learning mode, years of study, instrument type, performance format, and weekly practice hours), while means and standard deviations are reported for continuous variables. Total and subscale scores of the K-MPAI-A^4^ have been calculated in accordance with the instrument guidelines. Internal consistency was assessed using Cronbach's alpha. Descriptive analyses have also been conducted to examine adolescents' self-reported anxiety experiences and help-seeking behaviors. Inferential analyses include independent-samples *t*-test, analysis of variance (ANOVA), Pearson correlation, multiple linear regression analyses and Chi-square test. A repeated-measures ANOVA was conducted to compare mean scores across the four K-MPAI-A dimensions, followed by Bonferroni-adjusted pairwise comparisons. Independent-samples *t*-test, simple and multiple linear regression analyses and one-way ANOVAs were conducted to examine whether demographic and learning-related variables predict K-MPAI-A scores, and whether significant statistical differences were found across these groups. K-MPAI-A total scores were entered as the dependent variable. Demographic characteristics were entered together in Model A, whereas learning-related variables were entered together in a separate Model B. Additionally, a chi-square test was conducted to examine the association between adolescents' help-seeking behaviors and perceived instrumental teacher support. Assumptions for regression analyses were checked, and effect sizes were reported where appropriate. Separate *t*-tests, ANOVAs, and simple regressions were used to examine bivariate differences of associations, while multiple regression was used to examine the combined contribution of relevant predictors.

## 3 Results

### 3.1 Learning-related characteristics

Over half of the adolescents in this sample had received formal instrumental education for 5–10 years (58.3%, *n* = 84), followed by more than 10 years (15.3%, *n* = 22). A few respondents reported 3–5 years (13.9%, *n* = 20) or 1–3 years of study (9.7%, *n* = 14), while 2.8% (*n* = 4) preferred not to disclose this information. Using ABRSM grade levels as a reference,^5^ nearly half of the respondents reported attaining Grade 8 (48.6%, *n* = 70). A further 23.6% (*n* = 34) were at Grade 6–7, and 16.7% (*n*=24) at Grade 4–5. Only 4.2% (*n* = 6) identified as Grade 1–3, while 6.9% (*n* = 10) preferred not to say. Most respondents were performing in both solo and ensemble settings (56.9%, *n* = 82), while almost a third mainly had solo performance experience (30.6%, *n* = 44). Ensemble-only experience was less common (10.4%, *n* = 15), and 2.1% (*n* = 3) preferred not to disclose their performance format. Western instruments were more prominently represented in this sample, particularly strings (31.9%, *n* = 46), keyboard (16.7%, *n* = 24), and woodwind (10.4%, *n* = 15). Chinese strings were reported by 9.0% (*n* = 13) of respondents, followed by Chinese plucked instruments (8.3%, *n* = 12) and Chinese wood instruments (5.6%, *n* = 8): Chinese percussion was the least frequently reported category (0.7%, *n* = 1). In addition, 6.9% (*n* = 10) of respondents indicated learning multiple Western instruments, 0.7% (*n* = 1) reported learning multiple Chinese instruments, and 4.2% (*n* = 6) reported learning both Western and Chinese instruments. The most common duration of practice reported was 1–3 h per week (45.1%, *n* = 65), followed by 3–5 h (22.9%, *n* = 33) and 5–10 h (16.0%, *n* = 23). Only 6.9% of respondents (*n* = 10) reported practice over 10 h per week, while 9.0% (*n* = 13) preferred not to disclose their weekly practice time. Finally, most respondents received one-to-one tuition (69.4%, *n* = 100), a smaller proportion of respondents reported engaging in two or more forms of instruction (22.9%, *n* = 33), while relatively few respondents reported experience with group lessons (5.6%, *n* = 8), classroom-based instruction (1.4%, *n* = 2), or other formats (0.7%, *n* = 1).

### 3.2 Distribution of K-MPAI-A scores

The overall K-MPAI-A scores reported by adolescents in this sample were low to moderate (*M* = 1.88, *SD* = 1.33). The mean scores and standard deviations for each item are shown in Table 2. The highest mean score was observed for item 12 (Just before I perform, I feel nervous) and the lowest mean score was recorded for item 1 (Before I perform, I get butterflies in my stomach). The scale demonstrated high internal consistency (Cronbach's α = 0.92; *N* = 144). At the subscale level, cognitive concerns appeared to be the most problematic aspect of MPA in this sample (*M* = 2.35), followed by somatic symptoms (*M* = 1.83), performance context (*M* = 1.74), and performance evaluation (*M* = 1.62). A repeated-measures ANOVA confirmed a significant difference across the four K-MPAI-A dimensions [*F*_(2.34, 334.13)_ = 18.67, *p* < 0.001, η*p*^2^= 0.115]. Follow-up Bonferroni comparisons showed that cognitive concerns were significantly higher than each of the other three dimensions (all *p* < 0.001). No significant differences were observed among the other three dimensions (all *p* > 0.05). These findings indicate that adolescents' cognitive concerns were more prominent than somatic symptoms, performance context, and performance evaluation.

**Table 2:** K-MPAI-A descriptive statistics.

Item	*M*	SD
1. Before I perform, I get butterflies in my stomach	1.15	1.56
2. I often worry about my ability to perform	2.04	1.81
3. I would rather play on my own, than in front of other people	1.94	1.87
4. Before I perform, I tremble or shake	1.86	1.89
5. When I perform in front of an audience, I am afraid of making mistakes	2.29	1.99
6. When I perform in front of an audience, my heart beats very fast	2.27	1.97
7. When I perform in front of an audience, I find it hard to concentrate on my music	1.21	1.49
8. If I make a mistake during a performance, I usually panic	1.80	1.76
9. When I perform in front of an audience, I get sweaty hands	2.06	1.98
10. When I finish performing, I usually feel happy with my performance^*^	1.95	1.83
11. I try to avoid playing on my own at a school concert	1.65	1.98
12. Just before I perform, I feel nervous	2.71	2.04
13. I worry that my parents or teacher might not like my performance	1.51	1.77
14. I would rather play in a group or ensemble, than on my own	1.63	1.92
15. My muscles feel tense when I perform	1.83	1.69

Note: ^*^This item was reverse scored before the descriptive statistics were calculated.

Source: The original K-MPAI-A (Osborne and Kenny, 2005). Written permission to use and translate the instrument was obtained from Professor Dianna T. Kenny.

### 3.3 Demographic and learning-related differences and predictors of K-MPAI-A scores

A series of simple and multiple regression analyses, independent-samples *t*-test, and one-way ANOVA were conducted to examine whether demographic and learning-related variables predicted adolescents' K-MPAI-A scores and whether results differed across groups. Respondents who selected “other” or preferred not to disclose their gender (*n* = 15) were excluded from analyses due to the small number of respondents in this group. An independent-samples *t*-test was conducted to assess differences in K-MPAI-A scores between boys and girls: girls tended to report higher scores than boys, but the difference did not reach statistical significance [*t*_(127)_ = −1.83, *p* = 0.069]. Next, simple linear regression analyses were conducted to examine whether age and gender separately predicted adolescents' self-reported K-MPAI-A scores. Older age was a significant positive predictor of K-MPAI-A scores [*F*_(1, 142)_ =23.96, *p* < 0.001], explaining 14.4% of the variance in K-MPAI-A scores (*R*^2^ = 0.144). In contrast, gender alone did not significantly predict K-MPAI-A scores [*F*_(1, 127)_ =3.35, *p* = 0.069], although girls tended to report higher K-MPAI-A scores than boys. To further examine whether gender and age predicted K-MPAI-A scores, demographic characteristics were then entered as predictors in the Model A: the overall model was significant [*F*_(2, 126)_ =13.91, *p* < 0.001], explaining 18.1% of the variance in K-MPAI-A scores (*R*^2^ = 0.181). Older age remained a significant positive predictor (β = 0.394, *P* < 0.001), and gender also became a significant predictor after controlling for age (β = −0.163, *p* = 0.046) with girls reporting higher K-MPAI-A scores than boys at comparable ages.

Self-reported music grade was significantly related to adolescents' K-MPAI-A scores. An independent-samples *t*-test showed that respondents in the beginner to intermediate group (Grades 1–5) reported significantly higher K-MPAI-A scores than those in the advanced to highly advanced (Grades 6–8) (*t*_(41.10)_ = 3.91, *p* < 0.001). In addition, an independent-samples *t*-test was conducted to investigate whether K-MPAI-A scores differed between adolescents who reported being familiar with solo performance and those who reported ensemble or mixed performance experiences. Solo performers reported significantly higher K-MPAI-A scores than ensemble performers and those who had both performance experiences [*t*_(139)_ =2.05, *p* = 0.042]. Similar to the group comparison, simple linear regression showed that performance format significantly predicted K-MPAI-A scores [*F*_(1, 139)_ = 4.21, *p* = 0.042, *R*^2^ = 0.029] indicating adolescents who reported solo performance tended to report higher K-MPAI-A than those who had experienced ensemble or mixed performance (*B* = −0.49, *SE* = 0.24, β = -0.17, *P* = 0.042). The assumption of homogeneity of variance was met (Levene's test, *p* = 0.055), so a one-way ANOVA was conducted to further examine differences in K-MPAI-A scores across Western instrument groups (e.g., strings, keyboard, woodwind, brass, percussion and mixed Western instruments). *Post hoc* comparisons using Tukey HSD indicated a significant difference between Western keyboard and Western woodwind players, with higher K-MPAI-A reported among keyboard players (*p* = 0.002) than woodwind. No other pairwise comparisons were statistically significant. In contrast, a one-way ANOVA examining Chinese instrument groups revealed no significant difference in K-MPAI-A scores. Tukey *post hoc* tests likewise indicated no significant pairwise differences. A multiple regression analysis was then conducted to examine whether learning-related variables predicted adolescents' K-MPAI-A score. In Model B, learning-related variables (e.g., self-reported music grades, instrument type, weekly practice hours, performance format, learning mode, and years of instrumental study) were added to the model. The overall model was statistically significant [*F*_(6, 116)_ = 3.49, *p* = 0.003] and accounted for ~15.3% of the variance in K-MPAI-A score. Performance format and instrument type did not remain significant in the combined model (*p* > 0.05 for both tests), though they significantly predicted K-MPAI-A scores in the separate analysis.

### 3.4 Self-reported experiences of nervousness, worry, or anxiety in music performance, help-seeking behaviors and perceived instrumental teacher support

Respondents were asked to report whether they have ever felt nervous, worried, or anxious when performing their instrument in front of others: Of the 143 respondents who answered this question, 40.6% (*n* = 58) selected “Yes,” 42.7% (*n* = 61) selected “Maybe,” and 16.8% (*n* = 24) selected “No”; one respondent did not provide an answer. This indicates that the majority of respondents have either experienced or were uncertain about experiencing performance-related worry, nervousness, or anxiety. Those who reported at least some relevant experiences (“Yes” or “Maybe”) were asked whether they have sought help from their instrument teachers to manage those feelings. Among the 119 valid responses, 5.9% (*n* = 7) indicated that they always sought help, with 12.6% (*n* = 15) most of the time, 14.3% (*n* = 17) about half of the time, and 47.9% (*n* = 57) sometimes seeking support; however, 19.3% (*n* = 23) reported never seeking help. All respondents were then asked whether their instrumental teachers have provided guidance to support confidence in performance. The majority (72.9%, *n* = 105) reported receiving such support, while 19.4% (*n*=28) indicated uncertainty (“Maybe”) and only 6.9% (*n* = 10) reported that they have not received this support.

Further analyses examined whether perceived teacher support was associated with K-MPAI-A scores and help-seeking behaviors. Adolescents' reports of receiving teacher support were associated with both MPA levels and help-seeking behaviors. Pupils who were unsure whether they had received support or reported no provided support showed significantly higher K-MPAI-A scores (*M* = 2.71, *SD* = 1.30) than those who definitely reported that they had received instrumental teacher support (*M* = 1.58, *SD* = 1.22, *t*_(141)_ = −4.79, *p* < 0.001, Cohen's *d* = 0.91). In addition, adolescents who reported that they have asked for help from their instrumental teachers were significantly more likely to report receiving support than those who did not seek help (χ^2^ (1, *N* = 119) = 11.80, *p* < 0.001, Cramér's *V* = 0.32). However, no significant association was found between adolescents' K-MPAI-A scores and help-seeking behaviors.

### 3.5 Adolescents' perceptions of instrumental teacher support

#### 3.5.1 Factors influencing adolescents' help-seeking behaviors

**Figure 1:**
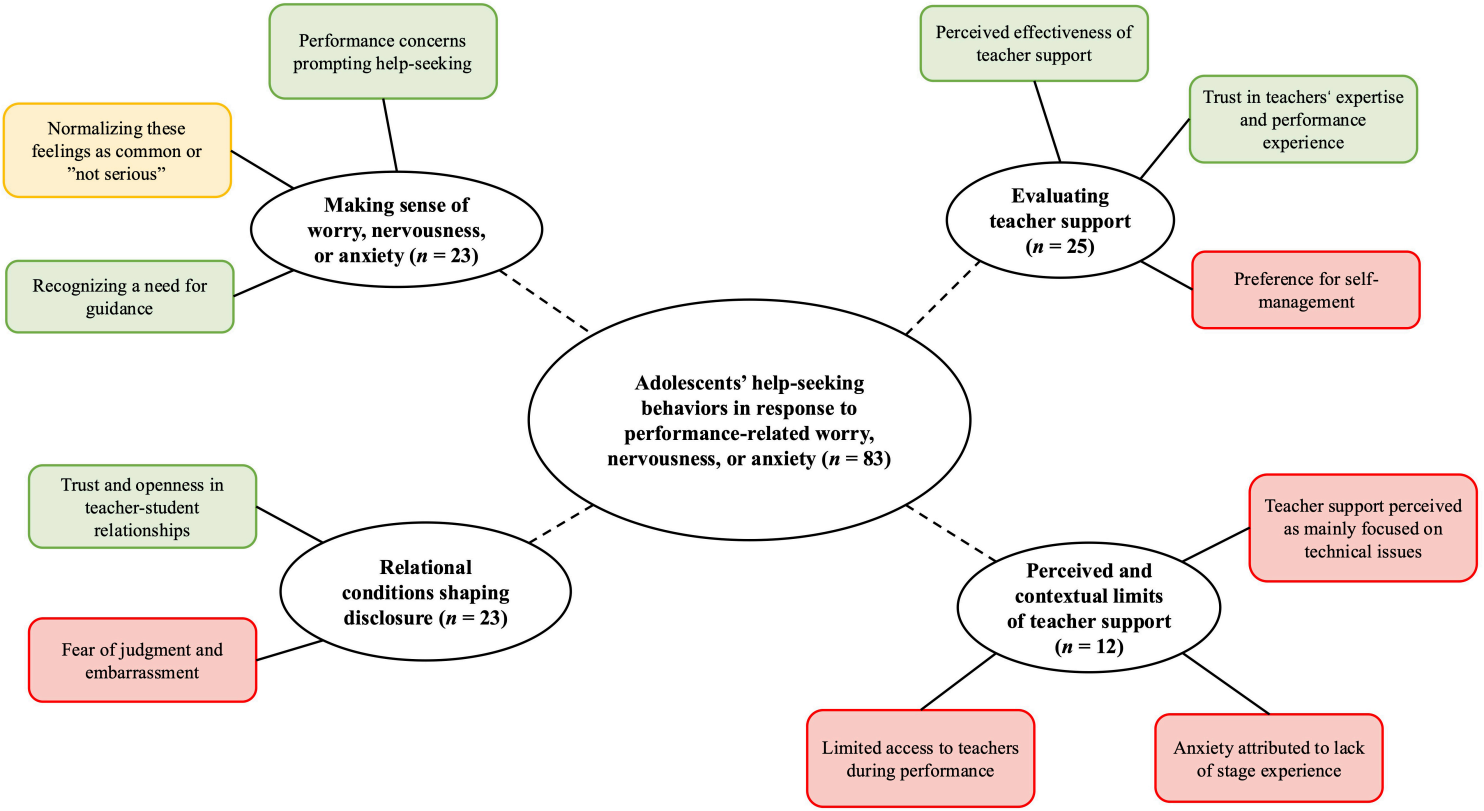
Thematic map of adolescents' help-seeking behaviors in response to the question about whether they have experienced nervous, worried, or anxious responses to performance (*N* = 83). Note: Green boxes indicate factors likely to encourage help-seeking; red boxes indicate factors likely to prevent help-seeking; yellow boxes indicate factors that may work in both directions.

Thematic analysis of adolescents' open-ended responses identified two patterns of help-seeking behaviors. Over half of respondents (*n* = 60) reported that when they felt anxious performing in front of others, they asked their instrumental teachers for help: as they explained, “I trust my teacher and know that they will help me” (R6) and “I hope to get useful suggestions from my teacher to overcome this issue” (R9). Within this group, a smaller subgroup of pupils (*n* = 12) described conditional or selective help seeking when the teacher was perceived as approachable and helpful: for example, one respondent mentioned, “I would ask for help if my teacher was available, but they are not always around when I need support” (R49). Some pupils (*n* = 23) reported that they would not seek help from their instrumental teachers when they felt anxious because they doubted the usefulness of teacher support, feared judgement and were embarrassed to disclose anxiety, or preferred to manage anxiety independently. They shared comments such as, “I did not think asking my teacher would help” (R52); “I am afraid that if I make a mistake, people will laugh at me and think I am not good enough, so I did not want to tell my teacher” (R55); and “I feel embarrassed about telling my teacher that I feel anxious” (R86); and “I can deal with it on my own” (R41). As illustrated in Figure 1, adolescents' help-seeking decisions are shaped by multiple dimensions, including: how they make sense of worry, nervousness, or anxiety; how they evaluate the need for support; their interpersonal experiences with teachers; and the contextual constraints on support-seeking they encounter in instrumental performances.

Adolescents' help-seeking behaviors were shaped by their appraisal and interpretations of worried, nervous, or anxious feelings experienced during performances. Performance-related concerns, such as fear of making mistakes or receiving negative evaluation, encouraged help-seeking behaviors when pupils recognized these worries as issues that could be addressed through their instrumental teachers' guidance, particularly in performance and examination settings. For example, one respondent explained that they asked for help because they were “afraid that I would play badly or make mistakes” (R18). Another described a strong pressure to meet high personal standards: “I want to be perfect and the best, but I am afraid of not reaching the goal” (R88). Similarly, one pupil said they were “very afraid of not performing well and disappointing others” (R98). Performance and examination contexts also seemed to intensify the need for support, as one respondent noted, “I asked for help because most of the time my performances have a specific purpose, such as assessments or meeting the requirements of music grades, and this makes me feel very stressed” (R102). Normalizing these feelings as common or “not serious” appeared to influence help-seeking behaviors in two different ways. Some respondents characterized anxiety as a manageable issue, but they still considered it necessary to ask for support in managing these feelings. For example, one pupil explained, “I thought it was only a small issue, but I still wanted my teacher to reassure me before the performance” (R125). However, others viewed these responses as not serious, which reduced their perceived need to seek help, as they felt that such feelings could be managed independently or would pass naturally. For example, students shared that “I did not think it was necessary to ask for help because the problem was not serious. I felt the anxiety would calm down on its own” (R32) and “It was not severe and I can adjust it by myself” (R41). Additionally, several respondents clearly recognized a need for teacher guidance when dealing with performance-related worry, nervousness, or anxiety. For example, some pupils described seeking advice because they wanted practical strategies to manage these feelings. One respondent explained, “I was looking for solutions and hoped my teacher could suggest effective ways to overcome my anxiety” (R35). Another pupil linked help-seeking to the possible impact of anxiety on performance, noting that “anxiety would affect my performance” (R105).

Adolescents also evaluated whether seeking instrumental teacher support was necessary. This evaluation was shaped by the perceived effectiveness of teacher support, their trust in teachers as professionals, or a preference for self-management. Many respondents reported that they had perceived effective teacher support and viewed instrumental teachers' professional knowledge and performance experience as valuable sources of assistance in these feelings: for example, one respondent explained, “My teacher can give me some useful suggestions” (R17). Another pupil similarly noted, “My instrumental teacher has a lot of stage experience, so they can give me reasonable advice based on my situation” (R135). However, other respondents preferred to rely on self-management or their own coping strategies, such as practicing more or regulating their feelings independently, and therefore did not always view instrumental teacher support as essential. As one pupil explained, “I felt the anxiety could ease if I worked through it myself, so I tried to regulate it on my own” (R105). Another respondent said, “I felt I could adjust it by practicing more” (R120).

Relational factors contributed to shaping whether adolescents were willing to disclose their anxious experiences. For several respondents, trust and openness in teacher-student relationships encouraged help-seeking, as they felt comfortable talking about these feelings and expected their teachers to respond with understanding rather than judging them. For example, one student explained, “Talking to my teacher and asking for help made me feel more relaxed” (R31). Another wanted them to understand the reason behind a weaker performance: “I wanted my teacher to know that sometimes I did not play well because I was nervous, not because I lacked ability” (R94). Similarly, one respondent said, “Talking to my teacher helped relieve my anxiety and the stomach cramps I felt when I was tense” (R28), while another noted, “My teacher made me feel safer and more confident to express these feelings” (R144). By contrast, some respondents described fear of judgment and embarrassment of disclosing anxious feelings as barriers to help-seeking. As one pupil explained, “I felt embarrassed to tell my teacher about my anxiety, and I was afraid others would laugh at me” (R55).

Perceived and contextual constraints also influenced adolescents' behaviors to seek help from their teachers, including the perception that teacher support mainly focused on technical issues rather than emotional needs, teacher responses that attributed anxiety to a lack of stage experience, and the absence of teachers during performance situations. A few pupils felt that when they expressed anxious feelings, their teachers responded primarily with technical advice. For example, one respondent said, “I felt my teacher was mainly guiding me on technical issues” (R97). Another explained, “I would not ask for help because my teacher thinks my anxiety is just because I do not have enough stage experience” (R67). Several respondents also described experiencing heightened or continuing anxiety while performing but having limited access to immediate support because their instrumental teachers were not present at that time. One pupil noted, “My instrumental teacher was not there during my performance” (R72). In these cases, even when teacher support might have been perceived as helpful, situational factors limited pupils' opportunities to seek help.

#### 3.5.2 Perceived strategies for confidence development and anxiety management

**Figure 2:**
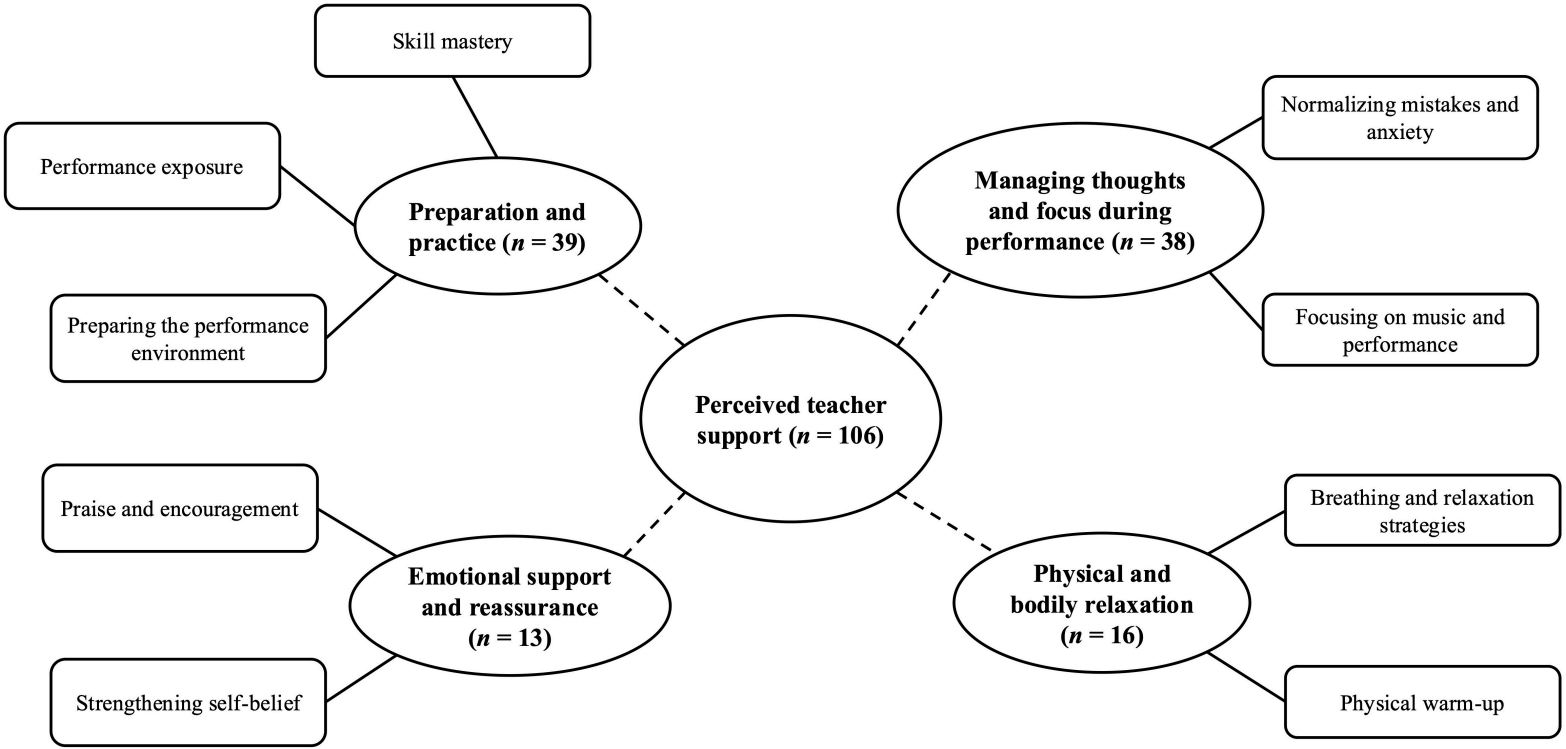
Reported instrumental teacher strategies for building performance confidence and managing anxiety. Note: The *n* values indicate coded responses rather than unique respondents.

Adolescent respondents shared a range of ways in which their instrumental teachers have supported them in terms of building confidence and managing anxiety (see Figure 2). These strategies demonstrate different kinds of support that students experienced in their lessons, performance preparation, and performance. The most frequently reported form of support related to preparation and practice (*n* = 39). Many respondents shared that they perceived their instrumental teachers as helping them feel more secure with the music before performance, including by strengthening technical skills and supporting familiarity with the repertoire. Some adolescents also referred to performance exposure, such as opportunities to play in front of family and friends, take part in competitions, record videos when performing, or wear concert attire when practicing performance before the actual event. For instance, a girl who reported relatively low K-MPAI-A scores and perceived instrumental teacher support described that “My teacher suggested getting used to the performance environment from time to time by recording videos, wearing formal costumes, and playing in front of my family and friends” (R8). In addition, several pupils mentioned that teachers help them prepare the performance environment: for example, one respondent shared that “My teacher helped me adjust the lights on stage to a comfortable brightness by talking to the technical team before performing…which really helped me concentrate on my performance” (R14). These findings suggest that adolescents described confidence as being supported not only through technical preparation but also through becoming more familiar with the conditions in which performance would take place.

Adolescent respondents also described ways in which their instrumental teachers guided them in managing their thoughts and focus during performance (*n* = 38). They reported that teachers encouraged them to shift attention away from mistakes, anxiety, audience evaluation, or potential outcomes of performance, and instead focus on the music or performance itself. Some respondents also described their teachers normalizing mistakes and anxiety, and trying to frame these as a manageable part of performance experiences. For example, respondents shared that “My teacher told me not to be afraid of making mistakes and focus on myself to make me feel more confident or less anxious when performing” (R6); “My teacher told me do not care about the outcomes…it is normal to feel anxious and make mistakes in musical performance…if you make a mistake somewhere, just skip it quickly” (R17); and “They told me that most of the audience just enjoyed my performance rather than judging me” (R34).

A smaller number of responses focused on physical and bodily relaxation (*n* = 16). Pupils mentioned deep breathing, relaxation strategies, and physical warm-ups, such as finger exercise, as approaches that instrumental teachers shared with them to regulate bodily tension before performance. Additionally, some students reported strategies regarding emotional support and reassurance from their instrumental teachers (*n* = 13), which helped them build confidence in performance. These methods involved teachers offering praise, encouragement, and effort to strengthen pupils' self-belief: for example, they described that “my teacher always encouraged me to believe in myself, like I am the best and it is my show” (R28).

#### 3.5.3 Evaluations of perceived instrumental teacher support

Respondents' evaluations of strategies provided by their instrumental teachers reflected varying levels of perceived helpfulness. Most responses described these strategies as helpful (*n* = 25): many respondents reported that they felt instrumental teacher support made anxiety feel more manageable and supported their confidence in performance. Some respondents explained that they felt more relaxed on stage, particularly when their teachers suggested adjustments to the performance environment that minimized visual distractions. Other respondents reported that their teachers' strategies were perceived as helping them relieve physical symptoms (such as stomach cramps) or noted that after trying the strategies they felt less anxious compared with previous performance experiences. Several pupils shared that strategies normalizing anxious or nervous feelings as a manageable part of music performance were perceived as helpful. At the same time, a few respondents (*n* = 9) acknowledged that the suggested strategies could make anxious responses feel more manageable but did not remove anxiety entirely (especially during performance), perceived strategies were difficult to apply in practice, or were less effective than their own coping methods. In contrast, a small number of respondents (*n* = 7) reported minimal or no benefit stating that the perceived strategies their teacher has shared did not work for them.

## 4 Discussion

### 4.1 Distribution of K-MPAI-A scores

The results indicate that negative feelings toward performance were common among adolescents aged 12 to 18 in this sample, with the majority reporting that they had felt nervous, anxious, or worried about performing in front of others. All respondents gave a non-zero K-MPAI-A score, although these responses may reflect either normal performance-related arousal or anxiety-related experiences. This finding aligns with earlier research suggesting that many adolescents report occasionally feeling nervous in lessons (Ryan et al., 2021) and MPA can begin early in a musician's development but is generally reported as normal or moderate rather than severe (Osborne and Kenny, 2005; Osborne et al., 2005; Tardif et al., 2024). These findings broadly support the current results, suggesting that performance-related nervousness and anxiety-related responses may be relatively common during adolescence, while previous studies have generally reported low-to-moderate rather than severe levels of MPA. However, the relatively low K-MPAI-A scores found in this study should not be seen as unimportant. They suggest that adolescence may be a key stage for recognizing and supporting performance anxiety before it becomes more persistent or disruptive. These findings point out the need for a balanced pedagogical response. Adolescents' anxious experiences should be taken seriously, as even moderate levels may affect their confidence, preparation, enjoyment, and willingness to perform, but such feelings should not automatically be pathologised. This interpretation is consistent with Herman and Clark's (2023) argument that MPA should be reconceptualised and de-pathologised rather than treated only as a problem to be eliminated. To support this distinction, throughout this paper we have referred to ‘lower and higher K-MPAI-A scores' rather than “low and high levels of MPA” to align with the salutogenic framing of the study and avoid the pathologisation of normal and manageable performance experiences.

Similar to the present study, Wang and Yang (2024) reported a moderate overall MPAI-A score (*M* = 40.82, *SD* = 12.51)^6^ among Chinese music high school students with the highest subscales score for the combined somatic and cognitive features dimension (they did not analyse these two aspects separately). A recent study by Ou and Qin (2025) found much higher MPA levels (*M* = 5.03, *SD* = 0.86) among their sample of 18–25 year old Chinese conservatoire undergraduate violin students using their translation of the K-MPAI-A than were reported by the 12–18 year olds in the current study. Kenny (2011) indicates that evaluative pressure and concerns about negative judgement are closely linked to heightened MPA. Research involving advanced student and professional musicians has also shown that performance anxiety remains a significant concern in more demanding training and performance environments (Papageorgi et al., 2013). Furthermore, Ou and Qin's (2025) study examined violin students from specialist music conservatories whereas the present study included adolescents learning a broader range of Western and Chinese instruments and was not limited to pre-professional training settings. This diverse sample may explain why total K-MPAI-A scores were lower in the present study.

Among the four K-MPAI-A dimensions, cognitive features showed the highest mean scores compared to somatic symptoms, performance context, and performance evaluation. This result indicates that anxiety was more frequently manifested as worries about performance, negative thoughts, and internal concerns: similar cognitive concerns were also evident in the qualitative findings (see section 3.5.1), where fear of mistakes and negative evaluation often motivated adolescents to seek support from their instrumental teachers. This finding is consistent with Osborne and Kenny's (2005) conceptualization of adolescent MPA as a multidimensional experience and indicates that cognitive concerns were particularly prominent in the present sample.

### 4.2 Demographic and learning factors related to K-MPAI-A scores

Age was found to be a significant predictor of K-MPAI-A scores in this sample, with older adolescents reporting higher levels of anxiety. This finding aligns with the rationale outlined earlier that adolescents' MPA may be shaped by developmental and self-evaluation concerns, including concerns about mistakes, performance outcomes, and evaluation (Kenny and Osborne, 2006; Osborne and Kenny, 2008; Patston and Osborne, 2016). It is also consistent with previous research indicating that MPA tends to increase with age in young musicians (Dempsey and Comeau, 2019; Nusseck et al., 2015; Osborne et al., 2005; Patston and Osborne, 2016; Papageorgi, 2022; Sârbescu and Dorgo, 2014). Chan's (2011) study of young orchestra musicians in Hong Kong reported a similar, although non-significant, pattern: older respondents tended to report higher levels of MPA than younger ones across both rehearsal and examination settings. The present findings extend this pattern by showing that age remained a significant predictor within this adolescent sample while differences in sample characteristics and musical learning contexts may help explain variations across studies.

No statistically significant differences were found between girls' and boys' K-MPAI-A scores in this sample and gender was not a significant predictor when it was entered into the regression model on its own. However, when age was included in the model, gender became significant with girls tending to report higher K-MPAI-A scores than boys after age had been taken into account. This suggests that gender may have a small association with K-MPAI-A scores in this sample, but that this association only became apparent when age-related variation was controlled. The non-significant group comparison may partly reflect the unequal sample size between girls and boys, which could have reduced the sensitivity of the comparison. It may also indicate that gender alone does not fully explain adolescents' K-MPAI-A scores. This finding partly aligns with previous studies reporting higher MPA among girls or female musicians (Osborne et al., 2005; Osborne and Kenny, 2005; Nusseck et al., 2015; Thomas and Nettelbeck, 2014; Tardif et al., 2024); however, it also resonates with students showing no significant gender difference, or no independent effect of sex once other variables were considered (Ryan, 2005; Papageorgi, 2022; Ou and Qin, 2025).

Adolescents who classified themselves as beginner to intermediate reported significantly higher K-MPAI-A scores than those who identified themselves as advanced to highly advanced. This finding differs from Papageorgi's (2007) research, which found that higher instrumental examination grade level was associated with higher levels of MPA among adolescent musicians. However, music grade in the present study was based on adolescents' self-reported classification according to ABRSM grade-level descriptions rather than verified examination records, so this variable may reflect not only formal attainment but also adolescents' perceived competence, confidence, perfectionistic tendencies and familiarity with musical learning demands. In this sample, pupils who saw themselves as more advanced may have felt prepared or capable in performance situations, which could help explain their reported lower K-MPAI-A scores. Alternatively, it may be indicating a ‘survivor bias' whereby those musicians who felt anxious and had not found ways to manage it stopped performing before they reached a higher standard. This possibility is worth considering given evidence that many young people stop taking part in musical activities during adolescence (Ruth and Müllensiefen, 2021), and that lower self-efficacy is associated with higher MPA levels (Bersh, 2022; Dempsey and Comeau, 2019) and career expectations among young musicians (Wang and Yang, 2024).

Since most respondents in this study played Western instruments (*n* = 103), the analysis focused mainly on these groups (e.g., strings, keyboard, woodwind, brass, percussion and mixed Western instruments). There was a notable difference between Western keyboard and woodwind players, with keyboard players reporting higher K-MPAI-A scores. This result contrasts with Yagisan's (2009) study and aligns more broadly with studies suggesting that MPA may vary by instrument type (e.g., Kaspersen and Götestam, 2002; Tardif et al., 2024). It may suggest that performance experiences vary across specific instruments rather than between broad instrumental categories. Instrument-related differences in K-MPAI-A scores may reflect the performance contexts and evaluative demands associated with particular instruments rather than instrument itself, which is consistent with Papageorgi's (2022) view that adolescent MPA is shaped by individual, task-related and environmental factors. In addition, a statistically significant difference of adolescent K-MPAI-A scores was found between solo performers and ensemble or those who had both performance experiences. Several previous studies have also reported higher K-MPAI-A scores in solo performance contexts than in ensemble settings (Brugués, 2011; Casanova et al., 2018; Papageorgi et al., 2013; Tardif et al., 2024). Learning-related variables (e.g., self-reported music grade, instrument type, weekly practice hours, performance format, learning mode, and years of instrumental study) were found to be significant predictors of adolescent K-MPAI-A scores. These variables contributed significantly to the regression model, suggesting that aspects of adolescents' musical learning experiences may be relevant to K-MPAI-A scores. Weekly practice hours and years of study reported by adolescents in this study did not significantly predict K-MPAI-A scores, which is consistent with previous research (Fehm and Schmidt, 2006; Kenny et al., 2013; Papageorgi et al., 2013; McCormick and McPherson, 2003); however, other studies have suggested that years of study may be related to MPA (Zakaria et al., 2013), indicating that the evidence remains mixed.

### 4.3 Perceived teacher support, help-seeking behaviors, and K-MPAI-A scores

The findings show that perceived instrumental teacher support was significantly associated with adolescents' K-MPAI-A scores. Pupils who were unsure whether they had received such support or reported no support showed higher K-MPAI-A scores than those who clearly reported receiving teacher support. This supports the view that teachers may play an important role in helping students manage performance anxiety (MacAfee and Comeau, 2023; Mazzarolo et al., 2023; Ryan et al., 2021; Sieger, 2017). The qualitative findings further suggest that instrumental teacher support may contribute not only to anxiety management but also to the development of confidence in performance situations. Pupils frequently described teachers as providing reassurance, encouragement, practical guidance, and performance preparation strategies, which may help them feel more capable and confident when performing. Recent evidence from Gill et al. (2026) supports this interpretation, showing that a teacher-guided programme enhanced adolescents' performance self-efficacy and confidence, alongside improvements in performance outcomes and some reductions in anxiety. This finding may also be considered alongside Wang and Yang's (2024) study of Chinese music high school students, which found that MPA was negatively associated with self-efficacy and future career expectations, though self-efficacy was not measured directly in the present study. Moreover, this reflects the salutogenic framing of the paper, as the study considers not only how MPA may be managed but also how instrumental teachers may be perceived by adolescents as supporting their confidence and positive performance experiences.

Adolescents' help-seeking behaviors are positively associated with perceived teacher support, indicating that pupils who reported that they have sought help from their instrumental teachers were more likely to report receiving support than those who did not ask for teacher help. This finding suggests that pupils' willingness to approach their teachers may be closely connected to whether teacher support is accessible, recognizable, or already experienced. This broadly aligns with research showing that many students would like more guidance from teachers on managing MPA (Studer et al., 2011), while teacher support for performance preparation and anxiety management may be present but not always consistently provided (Ryan et al., 2021).

No significant association between K-MPAI-A scores and help-seeking behaviors was found in the present study, suggesting that pupils with higher K-MPAI-A scores were not necessarily more likely to seek help from their instrumental teachers. Help-seeking behaviors may depend on how pupils interpret their anxious feelings and whether they believe teacher support is necessary. In this study, normalizing anxious experiences appeared to have a dual role: it may have helped some adolescents' view these experiences as manageable and easier to discuss with teachers, while leading others to see it as normal or not serious enough to require help. This interpretation broadly connects with Herman and Clark's (2023) argument that MPA should not be automatically pathologised but understood as a complex performance-related response that can be interpreted and managed in different ways. It also aligns with research highlighting the importance of open teacher-student communication in supporting students with MPA, while also recognizing that teacher support for anxiety management may not always be explicit or consistently provided in lessons (MacAfee and Comeau, 2023; Ryan et al., 2021). Theoretically, these findings contribute to a salutogenic understanding of adolescent MPA by suggesting that perceived teacher support may be relevant not only to anxiety management, but also to how adolescents understand confidence, preparation, and positive performance experiences. The findings also suggest that instrumental teachers may need to make support for performance confidence and anxiety management more explicit within lessons.

### 4.4 Perceived teacher strategies for confidence building and anxiety management

Findings from this study indicate that strategies identified by adolescent pupils are multidimensional and integrated into various aspects, as many respondents also mentioned more than one kind of teacher help. These results suggest that they experienced support as a combination of practical, cognitive, physical, and emotional guidance. From the perspective of the social support framework (Malecki and Demaray, 2003), these responses reflect informational, instrumental, emotional, and appraisal support, meaning that teacher support in this context may be understood as a broader supportive process for perceived anxiety management and confidence-building in musical performance.

Many adolescents described advice, tips, or guidance regarding building performance confidence and managing anxiety that they had received from their instrumental teachers, such as encouraging them to focus on the music itself, staying attentive during performance, and viewing mistakes or nervous feelings as more manageable. These approaches are broadly in line with cognitive and attention strategies discussed in previous MPA management research (e.g., Kaleńska-Rodzaj, 2023; Moura and Serra, 2021; Ryan et al., 2021). Preparation and practice were the main forms of support that adolescents in this sample reported receiving from their instrumental teachers. These approaches partly align with previous research supporting the significance of preparation and exposure-based methods in reducing performance anxiety and improving perceived control from both teachers and students' perspectives (e.g., Gill et al., 2024; Huang and Yu, 2022; MacAfee and Comeau, 2023; Moura and Serra, 2021; Norton, 2016; Ryan et al., 2021; Sieger, 2017). Recent findings by Mazzarolo et al. (2026) further support the value of exposure-based approaches, reporting that studio teachers frequently used simulated performance as a strategy for managing students' MPA, and that teachers perceived such exposure-based activities, including performing for peers or family members, informal stage performances, and recording, as helpful for building students' confidence and reducing the perceived threat of performance situations. Additionally, some respondents reported strategies regarding physical and bodily regulation, including deep breathing, relaxation techniques, and finger exercises, which are consistent with established approaches to managing somatic symptoms of MPA (e.g., Huang and Yu, 2022; MacAfee and Comeau, 2023; Moura and Serra, 2021; Ryan et al., 2021; Sieger, 2017).

Finally, some respondents referred to emotional support and reassurance from their instrumental teachers, including encouragement, comfort, and being helped to feel more confident before performance. This finding was supported by pupils' interpretation of help-seeking barriers, where a few reported that their teachers focused mainly on technical issues and paid limited attention to their emotional needs. These responses suggest that emotional support may be essential not simply as a separate anxiety-management technique, but as part of the relational context that makes adolescents more willing to disclose performance anxiety. In music learning contexts, Ryan et al. (2021) similarly highlight the relevance of the student-teacher relationship in adolescent pianists' performance preparation and anxiety experiences, while MacAfee and Comeau (2023) identify open communication as one of several strategies teachers use or discuss in supporting students with MPA. The psychological safety of a learning environment and its effects on confidence were mentioned by teachers in research by Norton (2016).

### 4.5 Perceived helpfulness and limitations of teacher support

Most respondents indicated that their instrumental teachers had provided support to enhance their confidence in instrumental performance, and many of those who reported receiving such support described the tips, advice, or guidance provided by their instrumental teachers as helpful in building performance confidence and managing anxiety. These findings are consistent with Norton's (2016) work on UK instrumental and vocal teachers' perspectives, which indicates that teachers perceive themselves as responsible for pupils' psychological performance-related health, and connect confidence-building to MPA management. Several teachers in that study also linked building pupils' confidence with decreasing anxious and nervous symptoms in performance contexts. The present study extends this teacher-focused perspective by showing that adolescent pupils also perceived teacher support as important for confidence building and MPA management. The quantitative results add further weight to this interpretation: adolescents who reported perceived instrumental teacher support for performance confidence building reported lower K-MPAI-A scores.

A smaller number of adolescents mentioned challenges in translating perceived instrumental teachers' advice into practice and perceived a mismatch in support. These results suggest potential gaps between teacher guidance and its practical application, and that some strategies (e.g., only focus on technical problems or attributed anxious feelings to lack of stage experience) did not match adolescents' emotional needs. Ryan et al. (2021) found that only 42% of piano students (*n* = 42) aged 11 to 17 reported that their teacher talked with them about how to deal with nervous feelings, and many of the provided strategies did not address the issue adequately. Tahirbegi (2022) also reported that some students said their instrumental tutors focused more on technical issues rather than MPA management and emotional aspects of music. This mismatch may also reflect the implicit nature of support within one-to-one instrumental teaching. Adolescents may not always know what effective support for performance anxiety should look like in practice, while teachers and students may have different expectations about whether managing anxiety in performance is mainly the teachers' responsibility, the students' responsibility, or shared between both. This uncertainty is relevant to wider MPA research, where music educators have been shown to use a range of strategies to support students, although research on how teachers address MPA in studio settings is still developing (Mazzarolo et al., 2023, 2026). Gaunt (2008) further shows that one-to-one instrumental teaching can involve intense and complex teacher-student relationships, shaped by trust, power and blurred supportive roles. Therefore, students' perceived mismatch in support may not simply indicate a lack of teacher concern, but may also arise from unclear communication, uncertainty about role boundaries, limited lesson time, and differences in teachers' training or confidence in addressing psychological aspects of performance. Students are also likely to be reluctant or uncertain about raising MPA directly with their teacher, particularly when lessons are perceived as focusing mainly on technical development (Tahirbegi, 2022; Ryan et al., 2021). These findings suggest that support for confidence building and anxiety management may need to be made more explicit, approachable and practical within instrumental lessons.

## 5 Practical implications

This study offers new insights into adolescents' individual perspectives relating to confidence and anxiety in musical performance and instrumental teacher support in China, which is a context that has not yet been adequately explored. Overall endorsement of the K-MPAI-A items was relatively low, although performance-related nervousness, worry, or anxiety was commonly reflected in both the K-MPAI-A responses and respondents' self-reports. This suggests that anxious feelings during performance may be a common but important part of adolescents' musical experience. Since adolescence is a key period for musical and personal development, these experiences may influence their confidence, motivation, and longer-term engagement with music. These findings have practical relevance for instrumental teachers, parents, adolescent pupils, and the wider field of Performing Arts Medicine and musicians' health and wellbeing. They highlight the importance of creating a positive educational environment that supports adolescents' musical achievement and emotional wellbeing. For instrumental teachers, the results suggest that pupils' performance-related anxiety should not be treated solely as a technical issue, or as a lack of stage experience that pupils need to manage alone. Paying attention to both emotional and technical aspects may make support more responsive to pupils' expressed needs. Although many adolescents in this study reported receiving instrumental teacher support in building performance confidence and managing anxiety, some pupils were unsure whether they had received such support, and others avoided asking for help because of embarrassment, fear of judgement, doubts about the effectiveness of teacher support or difficulty applying teachers' advice in practice. This suggests that teachers may need to make support more visible, approachable, and tangible in practice. Parents may also play a supportive role by recognizing that anxious feelings in performance are common, and by encouraging their children to seek help from their instrumental teacher. More broadly, this study contributes to the Performance Arts Medicine and musicians' health and wellbeing field by paying attention to performance-related anxiety before musicians enter higher education or professional training. Early and educationally grounded support may provide a valuable context in which adolescents can develop performance confidence, discuss performance experiences that they are worried about more openly, and remain engaged in musical learning and participation.

## 6 Limitations and future directions

The results of this study were based on adolescents' self-reported experiences of performance confidence/anxiety and their perceptions of instrumental teacher support. Their views may have been influenced by memory, social expectations, or individual interpretations. Perceived instrumental teacher support was also measured using study-specific survey items rather than a validated teacher-support scale. Therefore, the results reflect the support they perceived rather than direct evidence of the strategies instrumental teachers applied. The recruitment strategy and sample characteristics may also limit the generalisability of the findings. Respondents were recruited through institutions and organizations connected to the first researcher, which may have introduced selection bias. In addition, the high proportion of advanced learners and Western instrument players may have influenced the observed K-MPAI-A scores and learning-related findings; however, this pattern may partly reflect current instrument-learning preferences in China, where many parents and students favor Western instruments over traditional Chinese instruments (Guan et al., 2025). Self-reported music grade was also based on pupils' own classification using ABRSM grade-level descriptions rather than verified examination records, so it may reflect perceived competence, confidence, or self-efficacy as well as musical attainment. Another limitation is that the survey did not collect information about how long pupils had studied with their current instrumental teachers. The length of the teacher-student relationship may influence both adolescents' perceptions of support and their willingness to seek help, as trust and communication may develop over time. Further research could examine this factor more directly. A further limitation is that help-seeking was only asked of respondents who reported at least some experience of nervousness, worry, or anxiety in performance, so help-seeking was framed mainly through a difficulty-focused or pathogenic lens rather than through a broader salutogenic perspective that considers how pupils seek support to build confidence, prepare for performance and develop positive performance experiences. Future research could ask all respondents about the support they seek from instrumental teachers, including both anxiety management and confidence development, which would provide a more balanced understanding of instrumental teacher support as both a response to performance-related challenges and a proactive part of adolescents' musical development. The open-ended survey responses provide useful insights but follow-up interviews or focus groups with adolescents would provide a deeper understanding of how pupils interpret and respond to instrumental teacher support. Future research could also apply multiple sources of data, such as teacher interviews, lesson observations, parent perspectives, or performance-related assessments to enhance exploration of how support strategies are developed, communicated, and received in practice, and whether they meet pupils' emotional and performance-related needs.

## 7 Conclusion

This study provides valuable insights into Chinese adolescent musicians' experiences of confidence and anxiety in musical performance, and their views of perceived instrumental teacher support. Findings suggest that, while self-report of anxious responses to performance settings were common, overall K-MPAI-A scores were relatively low, with cognitive concerns appearing more prominent than somatic symptoms, and performance context and evaluation. Higher K-MPAI-A scores were associated with older age and lower self-reported music grade, while perceived teacher support was related to lower K-MPAI-A scores. Adolescent pupils who were only familiar with solo performance reported higher K-MPAI-A scores than those who had ensemble or mixed performance experiences. Most adolescents who had experienced worried, nervous, or anxious feelings about performance reported engaging in seeking help from their instrumental teachers and receiving some form of confidence-building support, but their help-seeking behaviors were shaped by perceptions of need, trust in teachers, fear of judgement, and the usefulness of instrumental teacher support. Such support was described not only in technical terms, but also through emotional reassurance, cognitive management, preparation strategies, and performance-related guidance. Overall, this study extends existing Chinese-context research on adolescent MPA by combining quantitative findings with qualitative responses, and adopting a salutogenic framing that considers confidence-building as well as anxiety management. The findings highlight that adolescents perceive instrumental teachers as potentially important sources of support for performance confidence and anxiety management, while also suggesting the need for support to be made more visible, approachable, and responsive to pupils' individual experiences.

## Data Availability

The datasets presented in this article are not readily available because they contain sensitive information from adolescent participants. Access is restricted due to ethical approval conditions, participant consent, and data protection requirements. Anonymised data may be made available from the corresponding author upon reasonable request, subject to ethical approval and appropriate data-sharing agreements. Requests to access the datasets should be directed to Yaxin Zhang, yaxin.zhang@york.ac.uk.

## Footnotes

1 The survey was open to adolescents aged 12-19 who were in full-time education and currently learning a musical instrument with at least one teacher. The upper age limit allowed for the possibility that some students in China may remain in secondary education until the age of 19 due to delayed school entry, interruptions to schooling, or returning to study; however, all eligible respondents in the sample were aged between 12 and 18.

2 Chinese-translated versions of the MPAI-A have recently been used in studies conducted in China (see Ou and Qin, 2025; Wang and Yang, 2024) but neither translated instrument was publicly available when data collection for the present study began.

3 The original English version was translated into Mandarin by the first researcher, and subsequently back-translated into English by an independent bilingual translator blinded to the original version. The back-translation version was compared with the original one, and minor revisions were made through discussion until equivalence was achieved. The remaining survey items, which were context-specific and not part of standardised instruments, were translated directly into Mandarin by the first researcher.

4 Osborne and Kenny (2005) organised 15 items of the K-MPAI-A into four dimensions: somatic symptoms (items 1, 4, 6, 9, and 15), cognitive concerns (items 2, 5, and 12), performance context (items 3, 11, and 14) and performance evaluation (items 7, 8, 10, and 13). Somatic and cognitive features describe the physical manifestations of MPA, worry and fear of making mistakes; Performance context refers to solo or group performances and nature of the audience; Performance evaluation represents evaluation from both audience and performer, and consequences stemming from those, particularly when a mistake is made (Osborne et al., 2005).

5 In the questionnaire, respondents were asked to self-report their approximate instrumental level using ABRSM grade levels as a reference. The response options were described as follows: Grade 1-3 (beginner to early intermediate) indicated that students were learning to play correctly and begin to add personal expression in simple music; Grade 4-5 (intermediate) described students as developing their technical ability, taking on more difficult music, and adding emotion and style to performance; Grade 6-7 (advanced) suggested that students were developing deeper musical understanding and making their playing more expressive and skilful; and Grade 8 (highly advanced) described students as refining their technique, developing deep musical understanding, and expressing emotion at a near-professional level.

6 These researchers used a five-point Likert scale, whereas the present study used the original seven-point response format, so the mean scores are not directly comparable.
